# *Gastrodia elata* BI.:A Comprehensive Review of Its Traditional Use, Botany, Phytochemistry, Pharmacology, and Pharmacokinetics

**DOI:** 10.1155/2023/5606021

**Published:** 2023-04-18

**Authors:** Ya-Nan Wu, Si-Hua Wen, Wei Zhang, Shang-Shang Yu, Kai Yang, Ding Liu, Chong-Bo Zhao, Jing Sun

**Affiliations:** ^1^Shaanxi University of Chinese Medicine, Xianyang 712046, China; ^2^Engineering Technology Research Center of Shaanxi Administration of Chinese Herbal Pieces, Xianyang 712046, China

## Abstract

**Materials and Methods:**

This article collects information from relevant documents, including scientific papers, books, and dissertations concerning *Gastrodia elata* BI.

**Results:**

To date, research on *Gastrodia elata* BI. has identified about 100 active compounds. Many compounds in *Gastrodia elata* BI. have biological activities, such as sedation and hypnosis, anticonvulsion, improvement of learning and memory, protection of neurons, antidepressive effects, lowering of blood pressure, promotion of angiogenesis, protection of cardiomyocytes, antiplatelet aggregation, anti-inflammatory activity, and amelioration of labor pains.

**Conclusion:**

Although many traditional uses of this plant have been confirmed, it is necessary to continue to study the relationship between its structure and function, clarify the mechanisms of pharmacological effects, and explore new clinical applications so as to better delineate the quality control standards for *Gastrodia elata* BI.

## 1. Introduction

As a traditional Chinese medicine, *Gastrodia elata* BI. (GB) has a long history, being recorded in many ancient books. The plant is mainly grown in Hubei, Sichuan, Yunnan, Guizhou, and Shaanxi [1]. There are nine preparations listed in the Pharmacopoeia of the People's Republic of China [2], namely, Tianma Pills, Tianma Headache Tablets, Tianma Gouteng Granules, Tianma Shouwu Tablets, Tianma Qufeng Patches, Tianma Xingnao Capsules, Bantian Ma Pills, Quan Tianma Capsules, and Strong Tianma Duzhong Pills. These preparations are used to treat headaches, chills, and nasal congestion, as well as dizziness, tinnitus, vertigo, tremor, insomnia, loss of memory, slow response, backache, epilepsy, convulsions, sore mouth, dry throat, hair loss, gray hair, chills, cold limbs, numbness, and other ailments. From the research on GB, about 100 active compounds have been isolated, and phenolic compounds, organic acids, steroids, polysaccharides, furan aldehydes, adenosines, and amino acids have been isolated and identified from the plant [3]. Moreover, many researchers have found that GB has a wide range of pharmacological activities, including static and hypnotic effects, anti-inflammatory and antioxidant effects, lowering of blood pressure, lowering blood lipids, and antiaging and antitumor effects.

**Figure 1:**
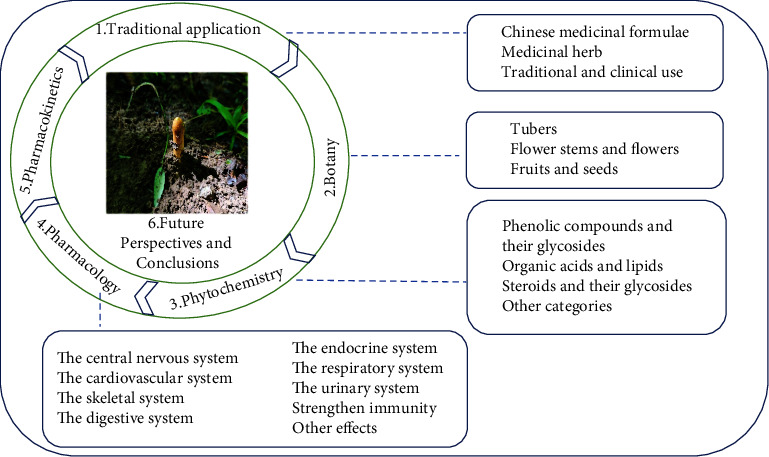
Article summary graph.

This article collects information from relevant documents, including scientific papers, books, and dissertations concerning GB. Some dissertations and scientific databases were used, including Baidu Scholars, Science Net, Weipu, Wanfang, and CNKI. We have systematically summarized many studies concerning GB, including its traditional uses, botany, phytochemistry, pharmacology, and pharmacokinetics. Finally, the problems and research directions of the GB Research Institute are discussed. The article summary chart is shown in Figure 1.

## 2. Traditional Application

GB is also known as Chijian, Dingfengcao, Guiduyou, and other names. The names are different in various historical periods. The earliest record is in the “Shennong's Classic of Materia Medica” [4], with “Chijian” as the correct name, also known as Limu or Guiduyou. The plants were used to kill ghosts, as a poison, in long-term service to benefit vitality, and for light body growth. In the Wei-Jin period, “WuPu Ben Cao” [5], Guiduyou was used as the correct name, with aliases Shencao and Yan Gouji. The plant was recorded in “Baopuzi” with Du Yaozhi as the correct name [6]. The earliest ancient book that mentions the name “GB” is “Mingyi Bielu” [7] in the late Han Dynasty, which says, “Wumuma, a name of Gastrodia.” During the Northern and Southern Dynasties, Lei Wei [5] first used GB as the correct name. At the end of the Sui Dynasty and the beginning of the Tang Dynasty, “Yao xinglun” [8] listed two items of Chijian and Chijianzhi, saying “Chijian, a GB, also known as Dingfengcao”, and the names in “Xinxiu Bencao” and “Shennong's Classic of Materia Medica” are consistent. The 2020 edition of “The Pharmacopoeia of the People's Republic of China” records that GB has the effects of dispelling wind and relieving spasms, suppressing liver yang, and dredging collaterals. It is used to treat convulsions in children, epilepsy, tetanus, headache, dizziness, hand and foot problems, numbness of the limbs, and rheumatic arthralgia.

Regarding whether GB and Chijian are the same thing, different generations of doctors held their own opinions. “Yaoxing Lun”, “Kaibao Bencao” in the Song Dynasty [9], “Jiayou Ben” [10], “Bencao Yanyi” [11], “Bencao Pinhui Jingyao” [12], and “Ben Cao Meng Yun” [13] believed that Chijian and Gastrodia were not the same thing. “Mengxi Bi Tan” [14], “Bencao Gangmu” [15], “Bencao Tongxuan” [16], and “Benzao Hui” [17] believed that the red arrow and GB were synonymous and were the same medicinal material.

At present, GB has a wide range of clinical uses. It is commonly used to treat infantile convulsions, epileptic convulsions, tetanus, headaches, dizziness, hand and foot problems, numbness of the limbs, and rheumatic arthralgia. The classic clinical prescriptions Tianma Alisma Decoction, Banxia Baizhu Tianma Decoction, and Tianma Gouteng Decoction can treat a variety of illnesses, including migraine, hypertension, atherosclerosis, and insufficient cerebral blood supply [4, 18].

It can be seen that the clinical application of GB is very extensive, and there are many prescriptions recorded in ancient Chinese literature. As shown in Table 1, the dosage forms include decoctions, pills, tablets, capsules, and granules. The extremely high nutritional value of *GB* makes its application in the beverage and health industry unique. The beverages and foods made with it have significant nourishing and strengthening effects [21–25].

## 3. Botany

**Figure 2:**
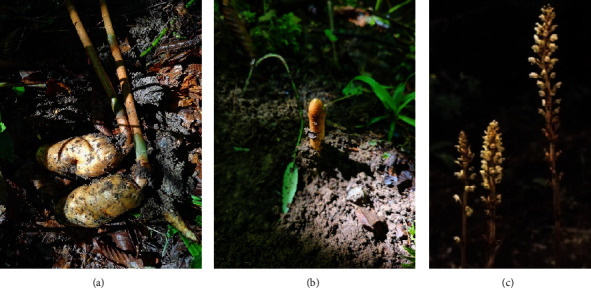
Plant GB: (a) tuber, (b) stem, and (c) flower.

GB is a saprophytic herb in the Orchidaceae *Gastrodia* genus, with a plant height of about 2 m. There are no roots and no leaves; only the above-ground flower stems and underground tubers that cannot conduct photosynthesis. The growth process requires fungal infection to provide nutrition. [26] The picture of GB is shown in Figure 2.

### 3.1. Tubers

According to the characteristics of different developmental stages, GB tubers can be divided into a protocorm, a vegetative propagation stem, a rice hemp, a white hemp, and a sisal hemp.

Protocorms are bulbs formed by the symbiotic germination of GB seeds, *Mycena osmundicola Lange, M. orchidicola* Fanet Guo, and other small mushrooms, with an average length of 0.4–0.7 mm and a diameter of 0.3–0.5 mm.

Vegetative propagation stems are formed by the differentiation and growth of protocorms, and these can also germinate through asexual reproduction of the white hemp and rice hemp [27–29].

Rice hemp refers to small tubers with a length of less than 2 cm formed by the growth of the apical or lateral buds of the vegetative propagation stem through sexual or vegetative propagation. Because it resembles a grain of rice, it is also called hemp, and is most suitable for asexual propagation and expansion [30].

White hemp refers to underground tubers with strong snow-white top buds. Small and medium white hemp can only be used for hemp seed cultivation and cannot be used as a medicine. Large white hemp can be used for both cultivation and as a medicine.

Sisal refers to the tubers of GB with terminal flower buds formed by the growth and reproduction of white hemp. It has the three characteristics of terminal flower stalk bud, tail umbilicus, and ring pattern around the body. It has a high content of active ingredients and is mostly harvested as commercial *GB*. [31].

### 3.2. Flower Stems and Flowers

The top bud of the sisal sprouts and grows to form a GB tuber. Its height is 0.5–1.3 m; the diameter is 1–1.5 cm, and there are generally 5–7 nodes. There are sheath-like phimosis membranous scales alternating on the nodes. The early stage of the flower stem is fleshy and solid, and the fruit is mature. The flower stem becomes hollow and the color becomes darker. The inflorescences of GB are racemes, which are mostly formed in the winter of the first year. The inflorescences are drawn out and bloom in the second year. Generally, each plant can have 30 to 70 flowers. The flowers are bisexual and symmetrical. The ovary and pedicel are composed of several parts, with various flower colors. Under natural conditions, GB relies on insect pollination. Both self-pollination and cross-pollination can produce fruits [32, 33].

### 3.3. Fruits and Seeds

The fruit of GB is a long oval capsule with a length of 1.5 to 1.7 cm and a diameter of 0.9 cm. It has six longitudinal ridges and is similar in color to the stem. Each fruit contains 10,000 to 50,000 seeds [31]. The seeds of GB are small and powdery. Under the microscope, the mature seeds are spindle-shaped, with a length of 0.8 mm and a width of 0.15–0.2 mm. The seeds have no endosperm and are composed of embryos and seed coats. The seed coats are white and translucent and are composed of parenchyma cells. The embryo is oval, light brown, or dark brown [32, 33].

## 4. Phytochemistry

### 4.1. Phenolic Compounds and Their Glycosides

#### 4.1.1. Phenolic Compounds Containing a Benzene Ring

**Figure 3:**
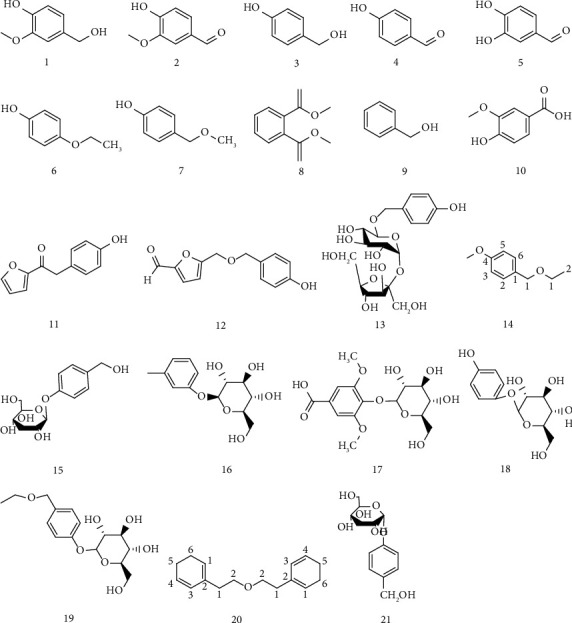
Phenolic compounds containing a benzene ring.

There are more than 40 phenolic compounds isolated from GB. The phenolic compounds containing a benzene ring are shown in Table 2 and the chemical structure is shown in Figure 3.

#### 4.1.2. Phenolic Compounds Containing Two or More Benzene Rings

**Figure 4:**
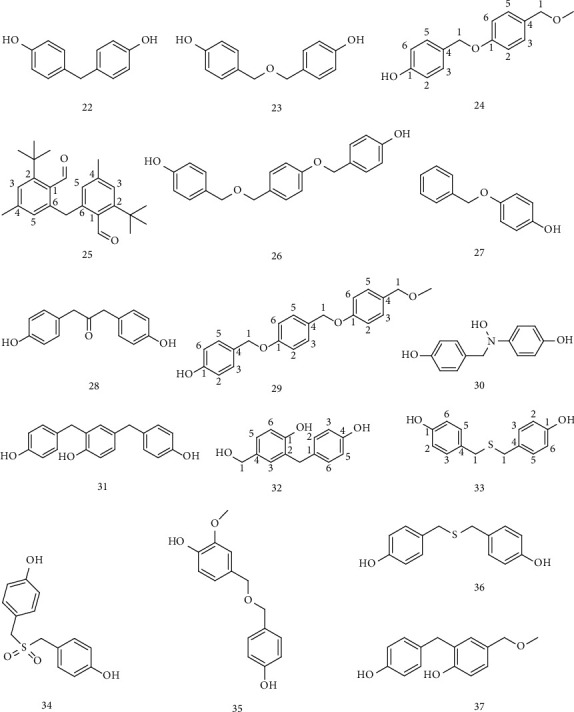
Phenolic compounds containing two or more benzene rings.

Phenolic compounds containing two or more benzene rings are shown in Table 3 and the chemical structure is shown in Figure 4.

### 4.2. Organic Acids and Lipids

**Figure 5:**
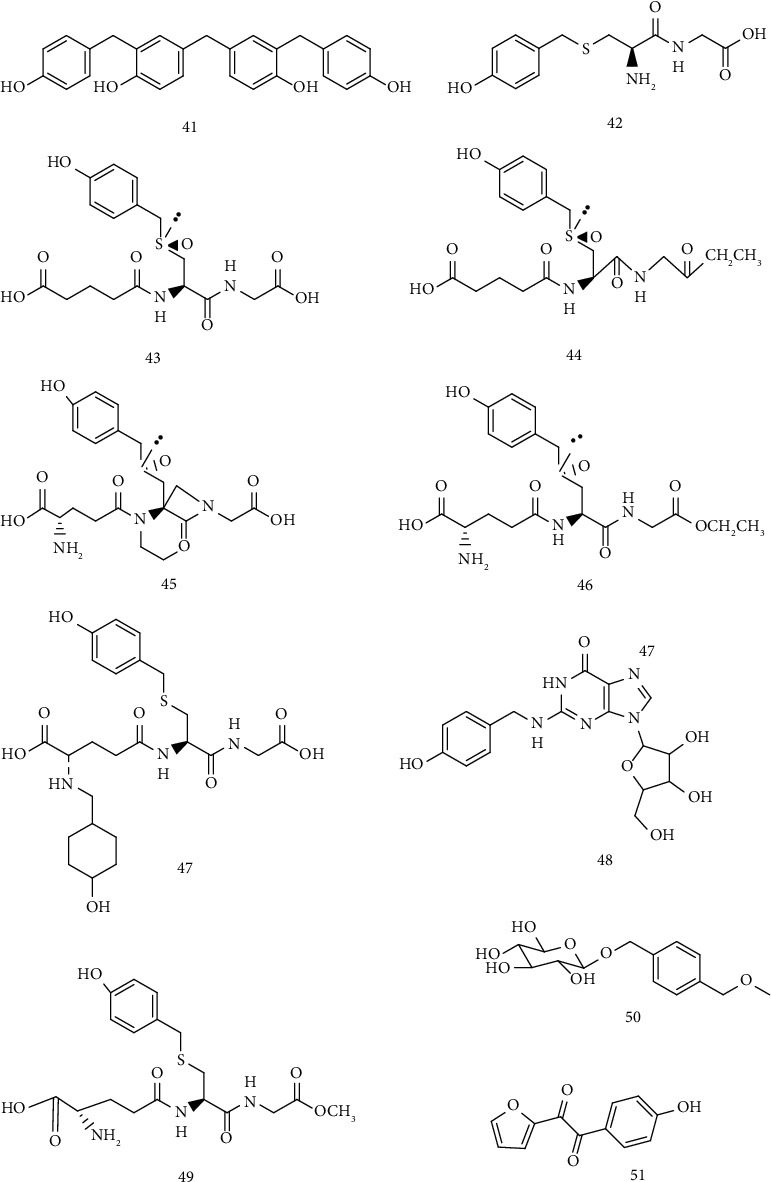
Organic acids and lipids.

The organic acids separated from *Gastrodia* are tabulated in Table 4 and the chemical structure is shown in Figure 5.

### 4.3. Steroids and Their Glycosides

**Figure 6:**
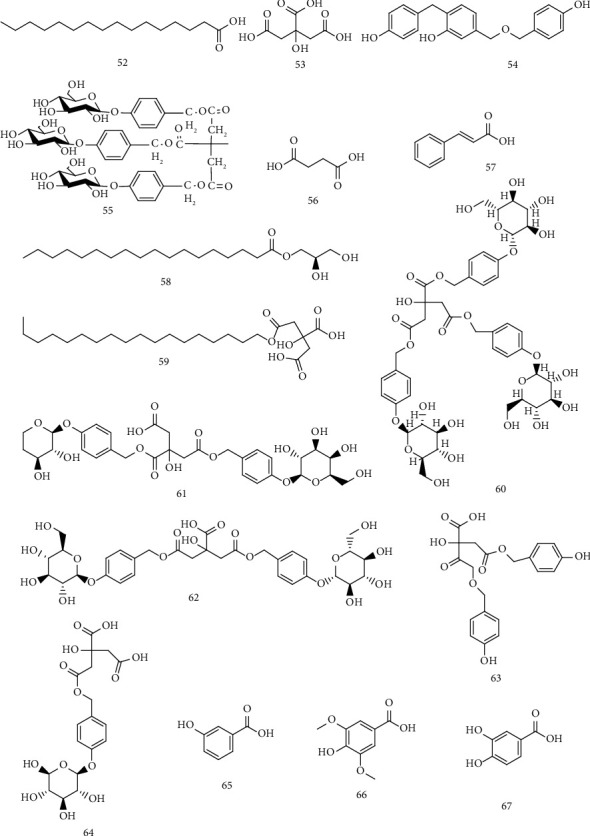
Steroids and their glycosides.

Five steroids have been isolated and identified from GB, as shown in Table 5 and the chemical structure is shown in Figure 6.

### 4.4. Other Categories

**Figure 7:**
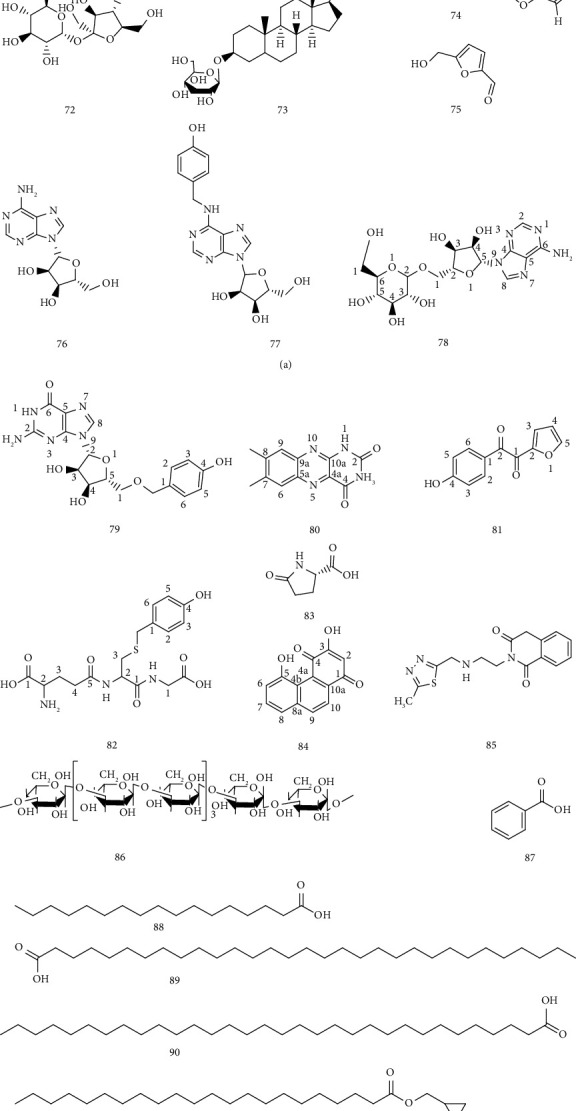
Other categories.

In addition to phenols, organic acids, and steroids, *Gastrodia* contains other compounds, including polysaccharides, furan aldehydes, adenosines, amino acids, and peptides. The specific ingredients are shown in Table 6 and the chemical structure is shown in Figure 7.

## 5. Pharmacology

**Figure 8:**
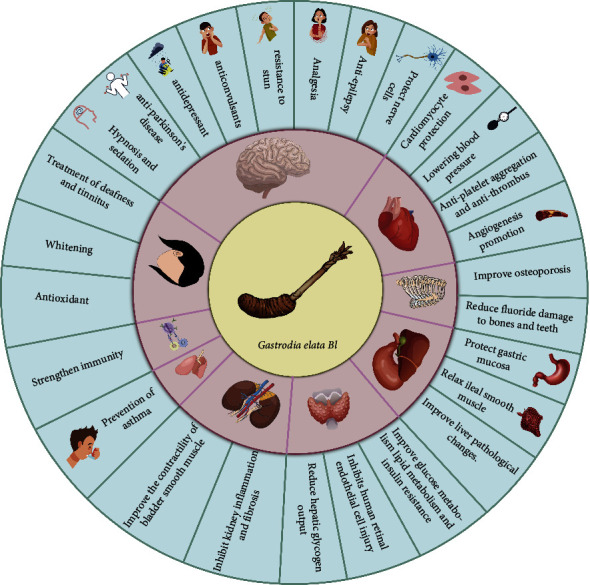
Pharmacological action diagram.

GB has a wide range of effects, including the central nervous system, cardiovascular system, skeletal system, digestive system, endocrine system, urinary system, and respiratory system. This is shown in Table 7, Figure 8.

### 5.1. The Effect of GB on the Central Nervous System

#### 5.1.1. Hypnosis and Sedation

GB has hypnotic and sedative effects [57]. Studies have shown that the main effect of fresh GB on sleep depends on the chemical composition of phenols [40], and gastrodin has a prominent effect among the phenols [59, 85]. The memory improvement caused by *Gastrodia* can ameliorate oxidative stress and boost neurotransmitter levels. The mechanism may be related to the up-regulation of central dopamine (DA) system activity, the regulation of dopamine receptor 2(D2)-mediated signaling pathways, and the regulation of monoamine neurotransmitters in the hypothalamus and hippocampus.

#### 5.1.2. Anti-Parkinson's Disease

GB also has significant effects on Parkinson's disease (PD), slowing the pathological process of Alzheimer's disease(AD) to a certain extent, reducing the deposition of beta-amyloid (A*β*), and improving learning and memory ability in AD dementia mouse models [60]. Studies have shown that GB extract can significantly improve the behavior of Parkinson's disease model mice [61], and *Gastrodia* extract can improve the cognitive dysfunction of PD rats [50, 61]. The decoctions had a therapeutic effect on transgenic Parkinson's mice [63]. The mechanism may be related to the enhancement of the human body's antioxidant capacity, protection of DA neurons in the brain, regulation of the level of monoamines in the brain, inhibition of a variety of apoptosis-related signaling pathways, activation of Wnt signaling pathways [65, 92], regulation of the Kelch-like epoxylopropylamine-related protein 1 (keap1)-nuclear factor E2 related factor2(Nrf2)/heme oxygenase-1(HO-1) pathway, or enhancement of the expression of downstream antioxidant genes and Superoxide dismutase(SOD) enzyme activity [66]. In addition, through studying the changes in the intestinal flora, three probiotics, *Lactobacillus johnsonii*, *Lactobacillus reuteri*, and *Lactobacillus murine*, were found in high doses of GB decoctions, each of which can help prevent and delay Alzheimer's disease. These findings present new ideas and methods [67].

#### 5.1.3. Antidepressant

*Gastrodia* extract has antidepressant effects [69]. Studies have shown that gastrodin can alleviate depression-like behavior in chronic unpredictable stress model (CUMS)-induced depressed rats. Gastrodin injection has also been used to treat patients with schizophrenia and immune dysfunction [70]. The antidepressant mechanism involves an increase in the monoamine neurotransmitters in the central nervous system, anti-inflammatory effects, increases in the number of new neurons, the rearrangement of the nerve cytoskeleton, and regulation of the expression of T helper cell 17 (Th17) and related inflammatory factors [71].

#### 5.1.4. Anticonvulsant

GB has anticonvulsant effects, and GB stalks and seeds also have good anticonvulsant effects [73, 102]. The mechanism of action is similar to that of carbamazepine.

#### 5.1.5. Antivertigo

Gastrodin injection has antivertigo effects and can effectively control acute vertigo [103]. It is effective in the treatment of post-traumatic vertigo [75]. Gastrodin had a significant effect on the treatment of middle-aged and elderly patients with vertigo [76].

#### 5.1.6. Analgesia

Gastrodin can effectively reduce pain and reduce the levels of serum inflammatory factors. Its mechanism of action may be related to the significant downregulation of c-fos gene expression in spinal dorsal horn tissue [72].

#### 5.1.7. Antiepileptic

*Gastrodia* has antiepileptic effects. Studies have confirmed that gastrodin can prolong the incubation period of generalized tonic-clonic seizure (GTCS) and minimal clonic seizure (MCS) in rats with pentylenetetrazole-induced epilepsy and improve cognitive function. The mechanism may be through regulating the abnormal expression of COX-2 [78], regulating the Nrf2/HO-1 classical antioxidant signal pathway, thereby reducing the expression of inflammatory factors iNOS [79], and regulating the level of monoamines in the brain to exert its antiepileptic effect [104], improve rat cognitive impairment, and protect nerves. Gastrodia can reduce the expression of serine-threonine protein kinase (p-AKT) and caspase 3 protein to resist the effect of resistance, thus triggering the model to play a protective role [80]. Gastrodin protected the brain of rats with pilocarpine-induced epilepsy by inhibiting the TLR4/NF-*κ*B signaling pathway [81]. Gastrodin injection inhibited the levels of proapoptotic factors in the cerebral cortex of rats with epileptic seizures after ischemic stroke, increased the levels of antiapoptotic factors, and reduced the level of p38 protein kinase in the body. It has the effect of protecting brain nerves and appears to be safe [50].

#### 5.1.8. Protects Nerve Cells

*Gastrodia* has a protective effect on nerve cells. An experiment compared the protective effects of GB powder and flour on nerve cells. The results showed that GB had a strong effect, and its mechanism of action may be related to the levels of 7-Aminobutyrate transaminase(GABA-T) mRNA and protein expression in the rat hippocampus [82].

### 5.2. Pharmacological Effects of Gastrodia on the Cardiovascular System

#### 5.2.1. Protects Cardiomyocytes

The effect of *Gastrodia* in protecting cardiomyocytes is mainly related to gastrodin. Gastrodin can inhibit the opening of mitochondrial permeability transition pore (mPTP) when cardiomyocytes undergo oxidative stress damage and thereby reduce apoptosis and reduce oxidative stress damage [83]. Gastrodin can also reduce autophagy, improve the clearance of autophagosomes, and reduce cell apoptosis [74]. Gastrodin upregulated the expression of 14-3-3*η* protein, inhibited cardiomyocyte oxidative damage [105], downregulated the degree of cardiomyocyte oxidative stress, reduced cell apoptosis, and acted as an anti-inflammatory [84]. These effects functioned to protect cardiomyocytes.

#### 5.2.2. Antihypertension

*Gastrodia* can effectively reduce hypertension caused by various factors, including essential hypertension [106], senile refractory hypertension [101], and spontaneous hypertension. The mechanism may be related to the inhibition of the release of vascular inflammatory substances *s* [20]. The results of a meta-analysis indicated that the blood pressure-lowering mechanism of gastrodin may be related to the involvement of 19 key target genes in 15 biological processes by influencing 14 hypertension pathways [107].

#### 5.2.3. Antiplatelet Aggregation and Antihrombosis

*Gastrodia* extract G2 had the effect of inhibiting platelet aggregation induced by adenosine diphosphate (ADP). *In vitro* experiments in rabbits demonstrated that the extract inhibited platelet activating factor (PAF)-induced platelet aggregation, confirming the antiplatelet aggregation effect of *Gastrodia* extract [86]. Experiments have examined the *in vitro* and *in vivo* activated partial thromboplastin timing and platelet aggregation rate induced by adenosine diphosphate as indicators to analyze the antiplatelet aggregation and antithrombotic effects of the drug, confirming that gastrodin can reduce platelet aggregation and thrombosis within a certain range [87]. The possible anticoagulant mechanism of gastrodin is related to its interference with the knob-hole interaction between fibrin molecules, which effectively inhibits the formation of blood clots and reduces the risk of thrombosis [88]. The ethyl acetate extract of *Gastrodia* significantly stimulated plasmin activity [108]. At the same time, phenolic compounds isolated from the methanol extract of *Gastrodia* had a strong inhibitory effect on platelet aggregation induced by U46619 [109].

#### 5.2.4. Promotes Angiogenesis

Gastrodiol components increased the expression of Vascular Endothelial Growth Factor Receptor 2 (VEGFR-2), *α*-SMA, and Smad-3 and reduce the expression of Ang-2 in the brain of middle cerebral artery occlusion/reper-fusion (MCAO/R) rats and promoted angiogenesis and maturation after cerebral ischemia [89]. Angiogenesis experiments with microvessels-deficient zebrafish showed that gastrodin significantly promoted angiogenesis [90]. Gastrodin promoted Vascular endothelial growth factor-A(VEGF-A) secreted by M2 macrophages to activate vascular endothelial cells and promote angiogenesis [110]. Experiments have shown that the ethanol extract of *Gastrodia* increased angiogenesis in a mouse lower limb ischemia model, and its mechanism may be related to the promotion of the expression of the pro-angiogenesis factor VEGF-A and its receptors VEGFR-2 and Angpt2 [35].

### 5.3. Skeletal System

Gastrodin can increase the proliferation of primary osteoblasts, activate the Nrf2/Keapl signaling pathway, reduce mitochondrial oxidative stress damage, maintain the steady state of mitochondrial membrane potential and the normal production of ATP to inhibit cell apoptosis, and promote the formation of osteogenic calcium nodules. These effects promote osteogenic differentiation and improve osteoporosis. The antioxidant capacity of rats was improved through treatment with different doses of gastrodin; the oxidative stress products and fluorine content of the body were reduced, and the damage of fluoride to bone and dentin was reduced to a certain extent. [100].

### 5.4. Digestive System

*Gastrodia* has a certain protective effect on the gastric mucosa [91], and at the same time, it has a relaxing effect on the smooth muscle of the ileum [45]. Gastrodin can prevent the loss of liver cell mitochondrial membrane potential caused by alcohol, reduce the release of cytochrome C in mitochondria, and inhibit the activation of caspase-3 in liver cells, thereby inhibiting liver cell apoptosis and returning abnormal liver function to normal. It can also effectively improve the pathological changes of the liver [92]. These studies show that *Gastrodia* can be used as an effective drug for the treatment of liver disease [93].

### 5.5. Endocrine System

Studies have shown that *Gastrodia* extract can improve glucose metabolism, lipid metabolism, and insulin resistance [94]. In type 2 diabetic rat animal models, *Gastrodia* significantly improved hypothalamic insulin signaling, enhanced insulin sensitivity, and reduced hepatic glycogen output in a hyper insulinemic state [95]. In addition, gastrodin (100 *µ*mol/L intervention for 24 h) had an inhibitory effect on human retinal endothelial cell damage induced by high glucose, and its mechanism may be related to the regulation of the Silent Information Regulator 1 (SIRT1)/TLR4/NF-*κ*Bp65 signaling pathway [64].

### 5.6. Urinary System

*Gastrodia* can effectively improve the contractility of bladder smooth muscle [97]. Studies have shown that gastrodin can reduce the levels of renal inflammatory factors and also inhibit oxidative stress by regulating Nrf2-mediated antioxidant signals and by activating AMPK. In addition, gastrodin inactivates the receptors of advanced glycation end products and the high mobility group box-1 (HMGB1) pathway and inhibits the activation of TLR, NF-*κ*B, and transforming growth factor-*β* (TGF-*β*). This suggests that gastrodin can inhibit carbon tetrachloride-induced renal inflammation and fibrosis through the AMPK/Nrf2/HMGB1 pathway [44].

### 5.7. Respiratory System

In IgE-mediated guinea pig asthma animal models, phenolic compounds extracted from *Gastrodia* (intervened at a dose of 12.5 mg/kg for 24 h) significantly inhibited the airway resistance in the acute and remission phases of asthma and effectively inhibited the recruitment of white blood cells, reduced histamine release, and inhibited eosinophil peroxidase (EPO) and phospholipase A activities. This suggests that *Gastrodia* extract may have certain clinical applications in the treatment of asthma [98].

### 5.8. Strengthens Immunity

Both the polysaccharides and water extracts of *Gastrodia* could promote the increase of mouse immunoglobulin levels and increase thymus and spleen indexes. In addition, *Gastrodia* injection improved the function of mouse phagocytes and serum lysozyme activity, enhanced the immune response and nonspecific effects of mouse T cells, and promoted the formation of specific antibodies, indicating that *Gastrodia* can enhance immunity [99].

### 5.9. Other Effects

#### 5.9.1. Antioxidant

The antioxidant capacity of rats was improved through treatment with different doses of gastrodin. The oxidative stress products and fluorine content of the body were reduced, and the damage of fluoride to bone and dentin was reduced to a certain extent. Reference [100]GB polysaccharides have a certain scavenging effect on ferrous ions, ABTS free radicals, hydroxyl free radicals, and DPPH free radicals. The scavenging effect is in the order of hydroxyl free radicals > DPPH free radicals > ABTS free radicals > metal ion free radicals [35]. Gastrodin has antioxidant and antiapoptotic effects in H_2_O_2_-induced oxidative stress damage. Gastrodin inhibits H_2_O_2_-induced oxidative damage and apoptosis of LSECs by activating the p38 MAPK/Nrf2/HO-1 pathway; it can reduce liver ischemia-reperfusion injury in mice through anti-inflammatory, antioxidant, and antiapoptotic effects [52]. *Gastrodia* extract can effectively improve the antioxidant capacity of rats, improve the level of oxidative stress-related indicators in rats, and thereby improve the hypoxia capacity of the rat body [53].

#### 5.9.2. Treatment of Deafness and Tinnitus

Gastrodin acupoint injection for sudden deafness accompanied by tinnitus can not only promote the disappearance of tinnitus but also improve the clinical effect [77]. Gastrodin injection is also used in the clinical treatment of patients with vertigo and tinnitus, with significant curative effect [101].

#### 5.9.3. Antitumor

Vascular dementia rats as experimental subjects were injected with GB extract, and the extract had a significant effect on improving the learning and memory of the mice. The main mechanism of action may be related to reducing oxidative damage in the hippocampus and scavenging free radicals [111]. Gastrodin significantly reduced the cerebral infarction volume and edema volume of rats with transient middle cerebral artery occlusion and significantly improved the neurological functions of patients [112]. In addition, gastrodin also inhibited neuronal apoptosis caused by glutamate and hypoxic-ischemic sugar and reduced the levels of nitric oxide and calcium ions in extracellular glutamate. Gastrodin also had an effect on the expression of aging-related genes in the brain tissue of rapidly aging mice. The antiaging effect of gastrodin is mainly through regulating the expression levels of some aging-related genes. The effective phenolic components in *Gastrodia* can reduce the area of infarcts in the whole brain and cortex, improve the distribution of neurons in the hippocampus and cortex of mice, reduce the activity of caspase-3, and enhance the expression of Bcl-2, confirming that gastrodin's neuroprotective effect is related to its mechanism of weakening the apoptotic pathway.

#### 5.9.4. Whitening

*Gastrodia* extract significantly reduced the melanin content in normal human melanocytes without obvious cytotoxicity. In addition, zebrafish *in vivo* experiments showed that *Gastrodia* extract effectively reduced melanin production without adverse side effects and no obvious cytotoxicity. This suggests that the extract of GB has a powerful whitening effect [113, 114].

## 6. Pharmacokinetics

In recent years, many domestic and foreign scholars have studied the pharmacokinetics of GB. The pharmacokinetics of gastrodin was studied by intragastric administration of gastrodin (100 mg/kg). The results showed that gastrodin could be detected in plasma at 4.98 minutes after administration. Tmax was (0.42 ± 0.14) h, and t1/2 was (1.13 ± 0.06) h [115]. The measured half-life differs in different species (The t1/2 of intravenous injection in rats, rabbits and dogs is 8.41 h, 38.4 h, 105 min respectively) [19]. Gastrodin can pass through the blood-brain barrier [116], and can also be metabolized to 4-hydroxy-benzyl alcohol to enter the blood-brain barrier to exert an effect on the central system [117], and finally be excreted through the bile [118]. The Tmax of 4-hydroxy-benzyl alcohol was 15 min, and the Cmax of plasma, bile, and brain were 109 ng/mg, 77.7 ng/mg, and 34.7 ng/mg [118]. Parishin is one of the active ingredients proven to have clinical efficacy. It is completely metabolized into gastrodin, 4-hydroxy-benzyl alcohol, parishin B and parishin C within 5 minutes in the body. Four metabolites are rapidly eliminated in the body [119]. N6-(4-hydroxybenzyl)-adenosine has obvious neuroprotective effect, Tmax is 69 min, t1/2 is 7.75 h [120]. 4-hydroxybenzaldehyde has protective effect on cerebral ischemia/reperfusion injury, in Rapid in vivo absorption, short half-life and low absolute bioavailability [121]. 4-Methoxybenzyl alcohol has a good brain protection effect, with a short half-life (t1/2 0.317 ± 0.094 h) [122]. GB extracts are mostly indexed by gastrodin and p-hydroxybenzyl alcohol. Other components in GB extract cause gastrodin and p-hydroxybenzyl alcohol to accumulate in tissues, with slow absorption and prolonged action. The Tmax of gastrodin is 70 min [123].

## 7. Future Perspectives and Conclusions

In summary, GB is a traditional Chinese medicine with a long history of use, and it is frequently employed in clinical practice. At present, many chemical components have been isolated and identified from this plant. There is no doubt that *GB* is an important Chinese medicine, and because of this, many professions have made significant contributions to the research on *GB*. However, in the research on *GB*, new problems and challenges continue to appear, and we need further research and exploration to meet the requirements of clinical use.

First, as a traditional Chinese medicine, GB has been studied more intensely in recent years, with more research being conducted on phenolic compounds, other compounds rarely being reported. Second, there are few studies on *GB* kinetics and toxicology. This aspect should receive more attention from researchers, and *in vivo* verification studies should be conducted to ensure drug safety. In particular, GB is used as medicine and food by villagers. It is commonly used to stew chickens, for example. The proper amount, effects of long-term use, and whether it can be toxic still need in-depth research. Third, when the GB medicinal materials are sold, they will be advertised as having a tonic effect that may be related to the pharmacological effects of GB such as antivertigo and enhancement of immunity, but whether there is actually a tonic effect and the specific pharmacological conditions still need in-depth research. Fourth, in China the wild resources of GB are declining, and the market resources are not in high demand. There are many kinds of “GB” in the medicinal material market. It is necessary to analyze and identify the various “GB” according to market conditions and identify those that can be used for medicinal purposes and those that can be used as health food. There are also some artificially cultivated *GB*. In view of the fact that there are many types and different quality of *GB* on the market, research on medicinal materials should be strengthened to ensure their quality.

In general, *GB* as a commonly used traditional Chinese medicine requires further research. This article systematically introduces the research status of GB at home and abroad in recent years, including traditional applications, phytochemistry, pharmacology, and pharmacokinetics. Although significant progress has been made, there are still problems associated with various aspects of the plant. This article also proposes some suggestions for solving these problems. Therefore, to further develop and utilize this Chinese medicine, we need to make continuous efforts in the future.

## Figures and Tables

**Table 1:** Traditional application and clinical use of *Gastrodia* in China.

Name	Medicinal herb	Traditional and clinical use	Ref
Run Ti Yuan	*Saposhnikovia divaricata* (Turcz.) Schischk (SDS)*, Cinnamomum cassia Presl* (CCP), *Boswellia carterii* Birdw (BCB), *Saiga tatarica Linnaeus* (STL), *Aconitum carmichaelii* Debx. (ACD), *Bombyx mori Linnaeus* (BML), *Areca catechu* L. (ACL), *Amomum kravanh Pierre* ex Gagnep (AKP), *Aquilaria sinensis* (Lour.)*Gilg* (ASG), *Tribulus terrestris* L. *Eugenia caryophyllata* Thunb. (ECT), *Vitex trifolia* L. var.*simplicifolia* Cham.:*Bos taurus domesticus Gmelin* (BTDG), *Pogostemon cablin* (Blanco) Benth., *Ephedra sinica Stapf* (ESS), *Rhinoceros unicornis* L., As_2_S_2,_*Moschus berezovskii Flerov* (MBF), *Aucklandia lappa* Decne (ALD), *Poria cocos*(Schw.) Wolf (PCW), TAG, *Notopterygium incisum* Ting ex H.T.Chang (NTC), *Bombyx mori* L., *Panax ginseng* C. A. Mey. (PGM), CCP, *Ligusticum chuanxiong* Hort., *White Tong Pearl Powder*, *Angelica pubescens* Maxim.f. *biserrata Shan et Yuan* (APM), *Buthus martensii Karsch* (BMK), *Pinellia ternata* (Thunb.) (PTT), ACD, *Bungarus multicinctus Blyth*, GB, *Succinum* (SUC), Hg, AKP, gold leaf	To cure all wind syndrome: paralysis of the limbs, mind stupidity, dysphasia, facial paralysis, spasm of the tendons, painful joints, dizziness, trance, forgetfulness, excess phlegm and saliva dullness, thick skin, and paralysis	Tai, 1996
Wuxi Yuan	*Atractylodes macrocephala* Koidz. (AMK), *Angelica dahurica* (Fisch. ex Hoffm) Benth.et Hook.f. (ADBH), *Zingiber officinale* Rose., *Citrus aurantium* L. (CAL), *Bambusa textilis McClure* (BTM), *Panthera tigris*, *Magnolia officinalis* Rehd. *et* Wils., *Polygonum multiflorum* Thunb. (PMT), *Mauremys reevesii*, *Tenodera sinensis Saussure*, *Amomum villosum* Lour.*, Vitex trifolia* L. var.*simplicifolia* Cham. ECT, BML, *Asarum heterotropoides*; Fr. Schmidt var. *mandshuricum* (Maxim.)Kitag.(AHFSVM), Ligusticum sinense Oliv.(LSO), S*ophora japonica* L.*, Equus asinus* L., *Citrus reticulata Blanco* (CRB), *Arisaema erubescens* (Wall.)Schott (AES), NTC, MBF, GB, PTT, PCW, APM, PGM, STL, *Pogostemon cablin* (Blanco) Benth., ACL, ACD, CCP, ASG, ESS, BML, TAG, BMK, SDS, *Bungarus multicinctus Blyth*, *Zaocys dhumnades* (Cantor), ALD, *Dendrobium nobile* Lindl., Hg, *Cicadidae*, *Ligusticum chuanxiong* Hort., AKP, S, ACD, CCP, HgS, As_2_S_2_, BTDG, *Vulpes*, Corvussp., Hg, ADS, *Gleditsia sinensis* Lam	To cure all wind syndrome: closed teeth, phlegm on the diaphragm, crooked mouth and eyes. It also cures paralysis, epilepsy, hand and foot twitching, restlessness, trouble walking, hemorrhoids, kidney wind poison, women's blood wind, dizziness and vomiting, skin swelling and itching, and pain all over the body	Tai, 1996
Mosuo Yuan	*Scrophularia ningpoensis* Hemsl.(SNH), *Sanguisorba officinalis* L., ACD, ALD, ECT, *Lindera aggregata* (Sims) Kosterm., BCB, As_2_S_2_, *Gleditsia sinensis* Lam., CCP, HgS, FeS_2_, MBF, GB, *White Tong Pearl Powder*	To cure apoplexy, paralysis, facial paralysis, lassitude, difficulty walking, and qi paralysis, and body aches and pains	Tai, 1996
Longnao Tianma Jian	*Cucumis melo* L.*, Lemna minor*, ACD, *Sanguisorba officinalis* L., SNH, GB	To cure all winds, paralysis, pain in joints, and upsurge of kidney wind poison, head and face weakness, swelling, tinnitus, hard of hearing, stuffy nose, dry mouth. It also cures the woman's blood and wind attack, body pain, dizziness, drowsiness, skin itching, rash and sores, and migraine headache	Tai, 1996
Niuhuang Xiao Wuxi Yuan	GB, ACD, *Sanguisorba officinalis* L., SNH	To cure all wind syndrome: numbness of the hands and feet, facial paralysis, dizziness, pain in the limbs, paralysis of apoplexy, epilepsy, face swelling and tinnitus, heavy pain, woman's blood winds, head spins and vomits, skin swollen and itchy, and body painful	Tai, 1996
Loujin Yuan	*Chrysanthemum lavandulifolium* (Fischer ex Trautvetter) Makino, *Astragalus membranaceus* (Fisch.) Bge. var. *mongholicus* (Bge.)*Hsiao* (AMBVM), LSO, BML, *Glycyrrhiza uralensis* Fisch. (GUF), NTC, ESS, PCW, *Paeonia lactiflora* Pall. (PLP), *Rhinoceros unicornis* L., ADBH, AHFSVM, PGM, SDS, *Ligusticum chuanxiong* Hort.*, Cinnamomum camphora* (L.)*Presl*, BTDG, MBF, TAG, BTM, *Bungarus multicinctus Blyth*, GB, *Rehmannia glutinosa* Libosch. (RGL), gold leaf	Cure winds syndrome, unconsciousness, stupefaction, language disability, Irritability and depression, facial paralysis, dizziness, headache, and pediatric epilepsy	Tai, 1996
Longhu Power	*Pharbitis nil* (L.) *Choisy, Pogostemon cablin* (Blanco) Benth., GB, *Achyranthes bidentata* Bl. (ABB), S, BTM, PTT, ACD, PMT, NTC, APM, *Bupleurum chinense* DC, *Ligusticum chuanxiong* Hort.*, Platycodon grandiflorum* (Jacq.)A.DC., *calcite calcitum*, *Illicium verum* Hook. f.*, Nardostachys jatamansi* (D. Don) DC., CCP, *Trogopterus xanthipes*, ADBH, *Chrysanthemum morifolium* Ramat.(CMR), ACD, BML, *Amomum villosum* Lour., Na_2_SO_4_·10H_2_O, ALD, Hg, As_2_S_2_, MBF, *Pheretima aspergillum* (E.Perrier) (PAE), *Zingiber officinale* Rose., HgS, *Tribulus terrestris* L., SDS, *Zaocys dhumnades* (Cantor), CCP	Hemiplegia, sores on the whole body, dizziness, facial paralysis, vomiting, and wet itching of the genitals	Tai, 1996
Shexiang Tianma Yuan	*Lemna minor*, ESS, SDS, GB, CMR, HgS, *Styrax tonkinensis*(Pierre) *Craib et* Hart., BCB, MBF, *Daemonorops draco* Bl.(DDB), *Sphora japonica* L	Treatment of wind, hand, and foot failure, lack of power to tremble, and whole body pain	Tai, 1996
Bafeng Power	3MgO·4SiO_2_·H_2_O, GB, CCP, MBF, BML, TAG, PTT, *calcite calcitum*	Cure various winds, headache, flushed face, dizziness, nasal congestion, dry throat, thick sputum and saliva, look drunk, joint pain, and tinnitus	Tai, 1996
Niuhuang Shengxi Yuan	*Plumbum Rubrum*, As_2_S_2_, Hg, STL, Hg, HgS, *Dens* Draconis., GB, Na_2_SO_4_·10H_2_O, PTT, *Bovidae*, CCP, BTDG	Cure wind and prosperous phlegm, headache and dizziness, trance, dry mouth and thirsty, restless sleep, and constipation	Tai, 1996
Chenshatianma Yuan	*Ligusticum chuanxiong* Hort., MBF, ADBH, HgS, TAG, GB, AES	Cure wind and prosperous phlegm, headache and dizziness, vomit, nausea, trance, forgetfulness, limb aches and tiredness, head and face swelling and itching, and numbness of hands and feet	Tai, 1996
Fangfeng Yuan	SDS*, Ligusticum chuanxiong* Hort., GB, GUF, HgS	Cure all wind, phlegm-heat, headache, nausea, dizziness, weakness of hands and feet, joint pain, language disability, trance, phlegm and salivation, drowsiness and forgetfulness, and sleep less	Tai, 1996
Datongsheng Baihuashe San	*Aralia chinensis* L., *Eucommia ulmoides* Oliv.(EUO), GB, BMK, *Prunus humilis* Bge., GB, ADS, *Magnolia officinalis* Rehd. *et* Wils., *Vitex trifolia* L. var.*simplicifolia* Cham., ALD, SDS, LSO, TAG, CCP, NTC, *Panthera tigris*, ADBH, *Dioscorea opposita* Thunb.*, Bungarus multicinctus Blyth,* CMR, ABB, GUF, CCO	Cure all winds, hands and feet twitching, Hemiplegia, mental dizziness, facial paralysis, language disability, excessive phlegm and salivation, skin Itching, pain in the joints, tinnitus, headache and dizziness; waist pain, swelling, itching, and sores	Tai, 1996
Hugu San	*Xanthium sibiricum* Patr.*, Drynaria fortunei* (Kunze) J. Sm., FeS_2_, DDB, TAG, PLP, ADS, CCP, ADBH, CMR, SDS, ABB, *Acanthopanax gracilistylus* W.W.Smith(AGS), GB, ACL, NTC, *Panthera tigris, Mauremys reevesii*	Cure wind, the evil energy enters between the skin and the bone marrow while the body is weak. Qi and blood fight. pain not in a fixed location, and Sleeplessness	Tai, 1996
Sijin Yuan	*Chaenomeles speciosa* (Sweet)*Nakai* (CSN), ABB, GB, *Cistanche deserticola* Y. C. Ma (CDM), ACD, *Panthera tigris*	Insufficient kidney meridian, legs and knees swelling and itching, inability to bend and extend, weak feet, inability to step on the ground, dull pain in the soles of the feet, lack of breath during walking, and weakness in the waist and knees	Tai, 1996
Ruxiang Yingtong Yuan	Os Draconis, *Scolopendra subspinipes mutilans* L. Koch, *Vigna umbellata Ohwi et Ohashi*, *Panthera tigris*, BML, *Aconitum kusnezoffii* Reichb. (AKR), *Liquidambar formosana Hance*, GB, ABB, ADS, BMK, BCB, *Momordica cochinchinensis* (Lour.) Spreng	Cure all wind, paralysis, Injured due to falling or being hit, and renal wind toxin	Tai, 1996
Zhuifeng San	ACD, SDS, *Ligusticum chuanxiong* Hort.*, BML*, *Schizonepeta tenuifolia* Eriq. (STE), CaSO_4_ · 2H_2_O, GUF, TAG, NTC, BMK, ADBH, AES, GB, PAE, BCB, AKR, CMR, As_2_S_2_	Headache. Long-term weakness of the liver, deficiency of qi and blood, and upward wind toxin, and feminine blood wind	Tai, 1996
Jufeng Power	*Piper longum* L., SDS, *Ligusticum chuanxiong* Hort., AHFSVM, GB, GUF,	Cure all winds, headache, and stuffy nose	Tai, 1996
Jingqi Yuan	*Perilla frutescens* (L.) Britt., CRB, ALD, ACD, ESS, *Bungarus multicinctus Blyth*, BML, AES, GB, HgS, BMK	The heart is affected by wind evil, trismus, drooling, coma, dementia when awake	Tai, 1996
Huantui Yuan	*Coix lacryma-jobi* L. var. *mayuew* (roman.)*Stapf, Photinia serrulata* Lindl*, Dendrobium nobile* Lindl., *Dioscorea septemloba Thunbt,* ABB, AES, NTC, SDS, AMBVM, ADS, GB, *Dipsacus asper* Wall. *ex* Henry, ACL, CSN	Cure the deficiency of the three-yin meridian of the feet, it is the attack of wind, cold, heat, and dampness	Tai, 1996
Mifang Huantui Yuan	*Coix lacryma-jobi* L. var. *mayuew* (Roman.)*Stapf, Photinia serratifolia* (Desfontaines) *Kalkman,* AES, ABB, CCP, ADS, GB, ACD, NTC, SDS, *Dendrobium nobile* Lindl.*, Dioscorea hypoglauca Palibin,* AMBVM, *Dipsacus asper* Wall. *ex Henry Atractylodes lancea* (Thunb.), ACL, CSN	Treats weakness of the kidney meridian, soreness and weakness of the waist and knees, or wind-cold, difficulty moving inability to flex, stretch, and dull pain in the soles of the feet, and wet and dry feet	Tai, 1996
Huatan Yuhu Yuan	AES, PTT, GB, *Pulsatilla chinensis* (Bunge) *Regel*	Cure wind phlegm, vomiting, headache, dizziness, fullness of the chest and diaphragm, indigestion, cough, phlegm, vomiting, and salivation	Tai, 1996
Wennaqi Yuan	*Callorhinus ursinus Linnaeus,* NH_4_Cl, *Capra hircus Linnaeus,* ASG, *Massa Medicata Fermentata*, [Ca_2_Mg_5_(Si_4_O_11_)OH_2_], PGM, *Psoralea corylifolia* L., CaCO_3_, *Morinda officinalis How, Ligusticum chuanxiong* Hort.*, AKP, Perilla frutescens* (L.) Britt., CAL, ALD*, Litsea cubeba* (Lour.)Pers.*, Trigonella foenum-graecum* L., GB, CRB, ECT, *Illicium verum* Hook. f., CCP, ACL, *Tribulus terrestris* L., ACL, *Dioscorea opposita* Thunb., CDM, AKP, ACD	Tonifying deficiency and replenishing qi, warming the back and removing pathogenic factors, nourishing essence, strengthening the spleen and stomach, promoting diet, and improving complexion	Tai, 1996
Mugua Yuan	*Cibotium barometz* (L.)J.Sm. (CBJS), *Artemisia argyi* Lévl. et Van., CSN, GB, ADS, *Dioscorea hypoglauca Palibin,* CDM, ABB	Cure kidney meridian weakness, heaviness of waist and knees, swelling and itching of legs and feet, injection sores, dull pain in soles of feet, constriction of muscles and veins, difficult walking, dark complexion, constipation, and diet reduction	Tai, 1996
Lurong Sijin Yuan	CDM, GB, *Cervus nippon Temminck, Cuscuta australis* R. Br., RGL, ABB, EUO, CSN	Treats weakness of muscles and bones due to liver and kidney deficiency, and internal heat	Tai, 1996
Congrong Dabu Yuan	ALD, ACD, *Illicium verum* Hook. f., CDM, *Zanthoxylum schinifolium* Sieb.*et* Zucc., *Morinda officinalis How,* ABB, *Tribulus terrestris* L.*, Prunus persica*(L.)*Batsch,* AMBVM, *Alisma orientate*(Sam.)Juzep., *Trigonella foenum-graecum* L.*, Schisandra chinensis*(Turcz.)Baill.(SCB0, ACL, GB, CCP*, Ligusticum chuanxiong* Hort.*, NTC*	Treats deficiency of blood and qi, bitterness of the mouth and dry tongue, thin limbs, and weakness of women	Tai, 1996
Yangshen San	BMK, GB, *Atractylodes lancea* (Thunb.), ACD, AKR	Cure kidney qi deficiency, pain in waist and foot joints, inability to flexion and extension of knees and tibia, and weak knees with chronic illnesses	Tai, 1996
Zhuxiang San	*Lindera aggregata* (Sims) Kosterm.*, Sparganium stoloniferum* Buch. -Ham.*, Curcuma phaeocaulis Valeton,* ADS, STE, GB, CCP*, Corydalis yanhusuo* W. T.*, Magnolia officinalis* Rehd. *et* Wils., ACD	Treat women with wind blood, dizziness, upset, hot hands and feet, irregular menstruation, and pain in the umbilical abdomen, less diet	Tai, 1996
Fanhun Power	*Angelica sinensis* (Oliv.)*Diels, Gleditsia sinensis* Lam.*, Zingiber officinale* Rose., CAL, AMK, PGM, ALD, PCW, ECT, *Magnolia officinalis* Rehd. *et* Wils., LSO, BTM, *Mauremys reevesii, Vitex trifolia* L. var.*simplicifolia* Cham.*, Tenodera sinensis Saussure,* PMT, ADBH, *Panthera tigris,* BML, *Amomum villosum* Lour., ESS, MBF, NTC, STL, PTT, ACD, SDS, *Bungarus multicinctus Blyth,* BML, ACL, TAG, AES, *Pogostemon cablin* (Blanco) Benth., *Equus asinus* L., *Dioscorea hypoglauca Palibin,* CCP, AHFSVM, CRB, *Sophora japonica* L.*, Zaocys dhumnades* (Cantor), ASG, BMK, APM, GB, HgS, Dendrobium nobile Lindl., As_2_S_2_, AKP, BTDG, CCP, Hg, ACD, *Cicadidae, Ligusticum chuanxiong* Hort., Corvussp., Hg, *Vulpes*, S, goldleaf	Treatment of children with various winds, epilepsy. Wind disease caused by chronic deficiency of various diseases, sleep more	Tai, 1996
Bazhen Power	GUF, GB, HgS, AES, BTDG, Hg, As_2_S_2_, *Cnidocampa flavescens Walker*[Monema flavescens Walker], Ag	Treat children with convulsions, fever, coma, vomiting, excessive phlegm, and salivation	Tai, 1996
Hujing Yuan	*Poria cum Radix Pini,* GB, Hg, BTM, *Picrorhiza scrophulariiflora Pennell*, HgS, MBF, TAG, AES, *Polygonum tinctorium* Ait.*, Quisqualis indica* L., *Cnidocampa flavescens Walke*r[Monema flavescens Walker]	Treat children with convulsions, lack of energy, sleep more, and wake up easily	Tai, 1996
Tianma Fangfeng Yuan	BML, BMK, GB, SDS, PGM, HgS, As_2_S_2_, MBF, GUF, BTDG	Treats convulsions, fever, excessive sleepiness, tetany, lack of energy, excessive phlegm, or wind-heat	Tai, 1996
Taiyi Power	AES*, Zaocys dhumnades* (Cantor), GB, ACD, ESS, BMK, TAG, BML, SUC, HgS, As_2_S_2_, GUF	Treats various winds in children, epilepsy, lack of energy, excessive phlegm and salivation, and asthenia wind	Tai, 1996
Zhisheng Baoming Power	BMK, TAG, AES, BML, HgS, MBF, SDS, GB, gold leaf, *Cryptotympana pustulata Fabricius*	Treats fetal and children fright, convulsions, and excessive phlegm, and salivation	Tai, 1996
Quanxie Guanyin San	*Nelumbo nucifera Gaertn, Dolichos lablab* L., PGM, *Massa Medicata Fermentata,* BMK, NTC, GB, SDS, ALD, ADBH, GUF, AMBVM, PCW	Treat children with exogenous cold, spleen and stomach internal injury, and promote diet	Tai, 1996
Huaijiaojian Pills	GB, *Ligusticum chuanxiong* Hort., GUF, *Dioscorea bulbifera* L., CMR, PGM, PMT, *Sophora flavescens* Alt., STE, SDS, *Sophora japonica* L	Sore	[19]
Tianma Gao	AKR, PCW, *Momordica cochinchinensis* (Lour) Spreng., GB, *Veratrum nigrum* L.*, Ligusticum chuanxiong* Hort.*, Stellera chamaejasme* L	Sore	[19]
Tianma San	*Veratrum nigrum* L., GB, *Stellera chamaejasme* L., ADBH, *Beckmannia syzigachne* (Steud.) Fern., PCW, AKR, *Dryopteris crassirhizoma Nakai*, AHFSVM, As_2_S_2_, Hg_2_Cl_2_	Sore	[19]
Shunfeng Yunqi San	AMK*, Lindera aggregata* (Sims) Kosterm., ASG, ADBH, *Perilla frutescens* (L.) Brit., CSN, GUF, CRB, GB, PGM	Smooth wind, smooth qi, hemiplegia	[20]
Zishou Jiefang Tang	NTC, SDS, ACD, *Ziziphus jujuba* Mill. var. *spinosa* (Bunge)*Hu ex* H. F. *Chou*, GB, CCP, STL, GUF	Dispel wind and resolve phlegm, strengthen healthy trends, and make language fluent	[20]
Banxia Tianma Baizhu Tang	PTT*, Hordeum vulgare* L., CRB, AMK, *Massa Medicata Fermentata*, GB, *Atractylodes lancea* (Thunb.), PGM, AMBVM, PCW, *Alisma orientate*(Sam.)Juzep.*, Phellodendron chinense* Schneid.*, Zingiber officinale* Rose	Invigorating the spleen and transforming the drink, calming the wind, and stopping dizziness	[20]
Baoan Wanling Pills	*Atractylodes lancea* (Thunb.), ESS, NTC, STE, SDS, AHFSVM, GB, BMK, ACD, AKR, *Dendrobium nobile* Lindl., PMT, HgS, ADS, *Ligusticum chuanxiong* Hort., GUF, *As*_*2*_*S*_*2*_	Dispel wind and dampness, activate blood and detoxify	[20]
Huichun Power	*Typhonium giganteum* Engl., As_2_S_2_, NTC, SDS, BMK, HgS, GB, BML, C_10_H_18_O, MBF, FeS_2,_*Fritillaria cirrhosa* D.Don, BTM, AES, BTDG	Clear heat and soothe the nerves, calm the wind, and resolve phlegm	[20]
Tianma Pills	GB, NTC, APM, EUO, *Cyathula officinalis Kuan, Dioscorea hypoglauca Palibin,* ACD, ADS, RGL, SNH	Dispelling wind and dampness, dredging collaterals and relieving pain, and replenishing liver and kidney	[2]
Tianma Toutong Tablets	GB, ADBH*, Ligusticum chuanxiong* Hort.*, Schizonepeta tenuifolia* Briq., ADS, BCB	Nourishes blood and dispels wind, disperses cold, and relieves pain	[2]
Tianma Gouteng granules	GB, *Uncaria rhynchophylla* (Miq.)Miq. ex Havil.(URMH), *Haliotis diversicolor Reeve, Gardenia jasminoides Ellis* (GJE)*, Scutellaria baicalensis Georgi, Cyathula officinalis Kuan,* EUO*, Leonurus japonicus* Houtt.*, Taxillus chinensis* (DC.) *Danser,* PMT, PCW	Pinggan and dispelling wind, clearing heat, and soothing the nerves	[2]
Tianma Shouwu Tablets	GB, ADBH, PMT, RGL, *Salvia miltiorrhiza* Bge.(SMB), *Ligusticum chuanxiong* Hort., ADS, *Tribulus terrestris* L.*, Morus alba* L.*, Eclipta prostrata* L.*, Ligustrum lucidum* Anit., PLP, *Polygonatum kingianum* Coll.et Hemsl., GUF	Nourish yin and kidney, nourish blood, and dispel wind	[2]
Tianma Qufengbu Talets	RGL, ADS, NTC, APM, ACD, CCP, GB, EUO, *Cyathula officinalis Kuan*, SNH, PCW	Warm the kidney and nourish the liver, dispel wind,, and relieve pain	[2]
Tianma Xingnao Capsule	GB, PAE, A*corus tatarinowii Schott, Polygala tenuifolia* Willd., RGL, CDM	Nourishes the liver and kidney, calms the liver and relieves wind, dredges the collaterals and relieves pain	[2]
Banxia Tianma Pills	*Pinellia ternata* (Thunb.)Breit., GB, AMBVM, PGM, *Atractylodes lancea* (Thunb.)DC., AMK, PCW, CRB, *Alisma orientale* (Sam.)Juzep., Massa Medicata Fermentata, *Hordeum vulgare* L.*, Phellodendron chinense* Schneid	To invigorate the spleen and dispel dampness, resolve phlegm, and dispel wind	[2]
Quantianma Capsule	GB	To calm the liver, relieve wind, and relieve spasm	[2]
Qiangli Tianma Duzhong Pills	GB, EUO, AKR, ACD, APM, LSO, SNH, ADS, RGL, *Cyathula officinalis Kuan, Viscum coloratum* (Komar.) *Nakai*, NTC	Dissipate wind and promote blood circulation, relax muscles, and relieve pain	[2]
Sha Yao	MBF*, Bufo bufo gargarizans* Cantor, C_10_H_18_O, *Rheum palmatum* L.(RPL), As_2_S_2_, *Atractylodes lancea* (Thunb.), ECT, GB, HgS, ESS, GUF	Eliminate heat and detoxification, open up filth, and resuscitation	[2]
Tianma Toutong Tablets	GB, ADBH, STE*, Ligusticum chuanxiong* Hort., ADS, BCB	Nourish blood and dispel wind, dispel the pain of cold	[2]
Tongtian Oral Liquid	*Ligusticum chuanxiong* Hort., GB, NTC, ADBH, PLP, CMR, *Mentha haplocalyx* Briq., SDS, AHFSVM, *Camellia sinensis* (L.) O. Ktze., GUF	Promoting blood circulation, removing blood stasis, dispelling wind, and relieving pain	[2]
Yianmashouwu Tablets	GB, PMT, RGL, *Eclipta prostrata* L.*, Ligustrum lucidum* Ait., *Polygonatum kingianum* Coll.et Hemsl., ADS, PLP, Morus alba L., *Tribulus terrestris* L., SMB, *Ligusticum chuanxiong* Hort., ADBH, GUF	Nourishes yin and kidney, nourishes blood, and extinguishes wind	[2]
An'Gong Jiangya Pills	BTDG*, Bubalus bubalis Linnaeus* (BBL), GB, *Coptis chinensis* Franch. (COP), *Scutellaria baicalensis Georgi*, GJE, *Curcuma wenyujin* Y. H. Chen et C.Ling, C_10_H_18_O, *Hyriopsis cumingii* (Lea), AMBVM, *Codonopsis pilosula* (Franch.)*Nannf, Ophiopogon japonicas* (L. f)Ker-Gawl., PLP, SCB*, Ligusticum chuanxiong* Hort	To clear away heat and calm, calm the liver, and suppress the yang	[2]
Qingxuan Zhitan Pills	GB, BML, BMK, PAE, *Pteria martensii* (Dunker)*, Cassia obtusifolia* L.*, Sophora japonica* L., BBL, BTDG, COP, *Scutellaria baicalensis Georgi*, SMB, *Ligusticum chuanxiong* Hort., PLP, ABB, CMR, DDB, *Crataegus pinnatifida* Bge.var. *major* N.E.Br., CCO, TAG, *Agkistrodon acutus* (Güenther), PTT, *Styrax tonkinensis*(Pierre) *Craib et Hart.*, C_10_H_18_O, PGM, AMBVM, AMK, PCW, *Ophiopogon japonicus*(L. f)Ker-Gawl., SNH, RGL, *Drynaria fortunei* (Kunze) J.Sm.*, Taxillus chinensis* (DC.)*Danser,* ASG, *Cyperus rotundus* L.(CRL), *Curcuma wenyujin* Y. H. Chen et C.Ling, CAL, *Pueraria lobata*(Willd)Ohwi (PLO), *Alisma orientale* (Sam.) Juzep	To calm the liver and eliminate wind, resolve phlegm, and dredge collaterals	[2]
Tianma Toufengling Capsule	GB, URMH, RGL, SNH, ADS, *Ligusticum chuanxiong* Hort., EUO, *Viscum coloratum*(Komar)*Nakai,* ABB, CMR	Nourish yin and suppress yang, dispel rheumatism, and strengthen muscles and bones	[2]
Zhennaoning Capsules	BBL, GB, LCH, AHFSVM, ADBH, PLO, LSO, Sus scrofa	extinguish the wind, clearing the meridians	[2]
Quantianma Capsule	GB	To calm the liver, extinguish wind, and relieve spasm	[2]
Qiangli Gastrodia Eucommia Capsule	GB, EUO, ABB, *Viscum coloratum*(Komar)*Nakai*, SNH, RGL, ADS, ACD, AKR, NTC, APM, LSO	To calm the liver and eliminate wind, promote blood circulation and dispel cold, relax muscles, and relieve pain	[2]
Tianshu Capsules	*Ligusticum chuanxiong* Hort., GB	Activating blood, calming the liver, dredging the collaterals, and relieving pain	[2]
Yianmu-Depressurization Tablet	GB, *Hyriopsis cumingii* (Lea), URMH, CMR, *Morus alba* L	To calm the liver and suppress the yang	[2]
Banxia Tianma Pills	PTT, GB, PGM, AMBVM, AMK, *Atractylodes lancea* (Thunb.), CRB, PCW, *Alisma orientate* (Sam.) Juzep.*, Massa Medicata Fermentata, Hordeum vulgare* L.*, Phellodendron chinense* Schneid	To invigorate the spleen and dispel dampness, resolve phlegm, and eliminate wind	[2]
Xingnao Zaizao Pills	AES, BML, TAG, C_10_H_18_O, *Acorus tatarinowii Schott,* AHFSVM, *Gleditsia sinensis* Lam., GB, PAE, BMK, *Pteria martensii* (Dunker)*, Haliotis diversicolor Reeve, Cassia obtusifolia* L.*, Panax notoginseng* (Burk.)F.H.Chen (PNC), ADS*, Ligusticum chuanxiong* Hort.*, Carthamus tinctorius* L., PLP, *Prunus persica*(L.)*Batsch,* PLO, AMBVM, PGM, AMK, *Lycium barbarum* L., SNH, PMT, *Epimedium brevicornu* Maxim., *Agrimonia pilosa* Ledeb., COP, *Forsythia suspensa* (Thunb.)*Vahl,* RPL, *Alisma orientate*(Sam.)Juzep.*, Stephania tetrandra* S. *Moore, Sophora japonica* L., ASG, ALD	Resolve phlegm and refresh the brain, expel wind, and activate collaterals	[2]
Zaizao Pills	PGM, AMBVM, AMK, PCW, PMT, RGL, ADS, SNH, *Chinemys reevesii* (gray)*, Drynaria fortunei* (Kunze) J. Sm.*, Taxillus chinensis* (DC.)*Danser,* C_10_H_18_O, MBF, BTM, BTDG, COP, HgS, BBL, CCO, *Panthera pardus* L., ADBH, NTC, SDS, ESS, AHFSVM, *Dioscorea hypoglauca Palibin, Agkistrodon acutus* (Güenther), PLO, *Anemone raddeana Regel, Pogostemon cablin* (Blanco)Benth., AKP, *Alpinia katsumadai Hayata*, ECT, ASG, *Santalum album* L.*, Lindera aggregata* (Sims) Kosterm., CRL, CRB, *Citrus grandis* “Tomentosa”, ACD, CCP, GB, BMK, BML, PAE, PNC, DDB, *Ligusticum chuanxiong* Hort., RPL*, Manis pentadactyla Linnaeus,* BCB, CMR, *Curcuma wenyujin* Y. H. Chen *et* C.Ling*, Pinus tabuliformis* Carr.*, Massa Medicata Fermentata*, GUF	To expel wind and phlegm, promote blood circulation, and clear collaterals	[2]
Bunao Pills	*Lycium barbarum* L., ADS, SCB, CDM, *Prunus persica* (L.)*Batsch, Alpinia oxyphylla* Miq.*, Platycladus orientalis* (L.)*Franco, Ziziphus jujuba* Mill. var. *spinosa* (Bunge)*Hu ex* H. F. *Chou, Polygala tenuifolia* Willd.*, Acorus tatarinowii Schott,* GB, *Os Draconis*, SUC, AES, BTM	Nourish essence and blood, soothe the nerves and invigorate the brain, resolve phlegm, and extinguish wind	[2]
Dahuoluo Pills	*Agkistrodon acutus* (Güenther)*, Zaocys dhumnades* (Cantor)., BMK, PAE, GB, CCO, AKR, CCP, AHFSVM, ESS, NTC, SDS, DDB, *Pogostemon cablin* (Blanco) Benth., AKP, BML, AES, BTDG, *Lindera aggregata* (Sims) Kosterm., ALD, ASG, ECT, CRB, CRL, MBF, *Styrax tonkinensis* (Pierre) *Craib et* Hart.*, C*_*10*_*H*_*18*_*O,, Anemone raddeana Regel*, PLP, CMR, BCB, DDB, COP, *Scutellaria baicalensis Georgi, Dryopteris crassirhizoma Nakai*, PLO, BBL, RPL, SNH, PGM, AMK, GUF, RGL, ADS, PMT, *Drynaria fortunei* (Kunze) J.Sm.*, Chinemys reevesii* (gray)*, Herbal Ephedrae*	Dispel wind and cold, remove dampness, resolve phlegm, activate collaterals, and relieve pain	[2]
Oriental Huoxue Plaster	ACD, AKR, AHFSVM, GB, BMK, NTC, APM, *Santalum album* L., BCB, CMR, *Carthamus tinctorius* L.*, Manis pentadactyla Linnaeus*, ADS, *Ligusticum chuanxiong* Hort., DDB, FeS_2_, *Momordica cochinchinensis* (Lour)Spreng., *Auricularia auricular* (L.)Underw. (AAU), C_10_H_18_O, CaSO_4_ • 2H_2_O, *Lonicera japonica* Thunb., F. *velutipes*, *Agaricus campestris,* [KAl(SO_4_)_2_ · 12H_2_O], *Herbal Ephedrae,* As_2_S_2_, *Acacia catechu* (L. f.)Willd.,	Dispel wind and cold, invigorate blood and remove blood stasis, relax tendons, and activate collaterals	[2]
Tianma Qfengbu Tablets	ACD, CCP, EUO, ABB, RGL, NTC, APM, GB, SNH, ADS, PCW	Warm the kidney and nourish the liver, dispel wind, and relieve pain	[2]
Zhikebao Tablet	*Aster tataricus* L. f.*, Platycodon grandiflorum* (Jacq.)A.DC.*, Peucedanum praeruptorum Dunn, Stemona sessilifolia* (Miq.)Miq., CRB, CRB, CAL, SCB, *Zingiber officinale* Rose.*, Papaver somniferum* L.*, Mentha haplocalyx* Briq., GUF, NH_4_Cl	lungs qi in Dispersion, relieve cough ,and relieve asthma	[2]
Shexiang Kangshuan Capsule	MBF, STL, AMBVM, *Siegesbeckia Pubescens* Mak.*, Lonicera japonica* Thunb.*, Spatholobus suberectus Dunn, Trachelospermum jasminoides* (Lindl.) Lem., RGL, ADS, *Carthamus tinctorius* L., PLP, *Zaocys dhumnades* (Cantor)., PAE, *Pueraria thomsonii* Benth., BMK, BML, *Whitmania pigra Whitman*, RPL, PNC, *Ligusticum chuanxiong* Hort., GB, AES	Clear the meridians, activate blood, refreshing brain, and removing blood stasis	[2]
Yinaoning Tablets	AMBVM*, Codonopsis pilosula* (Franch.)*Nannf*, PMT, *Ganoderma lucidum* (Leyss. ex Fr.) Karst. *Ligustrum lucidum* Ait.*, Yerbadetajo Herb, Taxillus chinensis* (DC.)*Danser,* GB, URMH, SMB, PLP, PAE, *Crataegus pinnatifida* Bge.var. *major* N.E.Br., SUC*, Hordeum vulgare* L	To invigorate qi and nourish the kidney, promote blood circulation, and dredge the meridians	[2]
Piantan Fuyuan Pills	AMBVM, PGM, ADS, RGL, AMK, PCW, *Alisma orientate* (Sam.)Juzep., AKP, *Ligusticum chuanxiong* Hort., PLP, SMB, PNC, ABB, GB, BML, BMK, URMH, TAG, PAE, PTT, *Gentiana macrophylla* Pall., CCO, SDS, EUO, *Psoralea corylifolia* L., *Drynaria fortunei* (Kunze) J.Sm., CRL, ASG, CAL, CCP, C_10_H_18_O, *Styrax tonkinensis*(Pierre) *Craib et* Hart.*, Ophiopogon japonicas* (L. f)Ker-Gawl., GUF	Invigorate qi and promote blood circulation, expel wind, and resolve phlegm	[2]
Renshen Zaizao Pills	PGM, AMBVM, AMK, PCW, PMT, ADS, RGL, *Chinemys reevesii* (gray)*, Panthera pardus* L.*, Taxillus chinensis* (DC.)*Danser, Drynaria fortunei* (Kunze) J.Sm., GB, AES, BML, PAE, BMK, BTM, PNC, *Ligusticum chuanxiong* Hort., PLP*, Curcuma wenyujin* Y. H. *Chen et* C.*Ling*, BCB, CMR, DDB, *Agkistrodon acutus* (Güenther), ADBH, NTC, CCO, ESS, SDS, PLO, *Dioscorea hypoglauca Palibin,* AHFSVM, ECT, *Lindera aggregata* (Sims) Kosterm., CRB, ASG, CRL, *Santalum album* L., *Alpinia katsumadai Hayata*, AKP, CRB, *Pogostemon cablin* (Blanco)Benth.*, Massa Medicata Fermentata,* ACD, CCP, MBF, C_10_H_18_O, HgS, SUC, BTDG, BBL, COP, RPL, SNH, GUF	Replenishing qi and nourishing blood, dispelling wind and phlegm, promoting blood circulation, and dredging collaterals	[2]
Kangshuan Zaizao Pill	*Whitmania pigra Whitman*, SMB, PNC, PAE, *Manis pentadactyla Linnaeus*, ABB, RPL, *Prunus persica* (L.)*Batsch, Carthamus tinctorius* L.*, Eupolyphaga sinensis Walker*, PLO, MBF, C_10_H_18_O, *Liquidambar orientalis* Mill., BTDG, AES, BMK, *Zaocys dhumnades* (Cantor)., GB, AHFSVM, *Dioscorea nipponica* Makino., CCO, PGM, AMBVM, ADS, PMT, HgS, *Alpinia katsumadai Hayata*, GUF,	Invigorate blood circulation, remove blood stasis, relieve muscles and collaterals, extinguish wind, and relieve spasm	[2]
Xizhi Luoda Capsules	*Siegesbeckia Herb, Whitmania pigra Whitman, Gentiana macrophylla* Pall., PNC, C_10_H_18_O, SMB, *Prunus persica* (L.)*Batsch, GB, Ligusticum chuanxiong* Hort.*, BTDG,* PTT, *Eupolyphaga sinensis Walker, Carthamus tinctorius* L., MBF, AES,	Resolving phlegm and promoting blood circulation, extinguishing wind, and dredging collaterals	[2]
Yangyin Jiangya Capsule	*Chinemys reevesii* (gray), PLP, GB, URMH, *Pteria martensii* (Dunker), Fe_2_O_3_, *Prunellavulgaris*L., *Sophora japonica* L., BTDG, C_10_H_18_O, PGM, SCB, RPL, CaSO_4_ · 2H_2_O, ALD, *Euodia rutaecarpa* (Juss.)Benth.,	Nourish yin and suppress yang, calm the liver, and soothe the nerves	[2]
Shixiang Fansheng Pills	*Liquidambar orientalis* Mill., MBF, *Styrax tonkinensis*(Pierre) *Craib et* Hart., C_10_H_18_O, *Santalum album* L., ALD, ASG, ECT, BCB, *Dalbergia odorifera* T. Chen*, Curcuma wenyujin* Y. H. Chen *et* C.Ling, CRL, BTDG, *vermiculite Schist seu Hydrobiotite Schist,* GB, BML, *Trichosanthes kirilowii* Maxim.*, Nelumbo nucifera* Gaertn., HgS, SUC, *Terminalia chebula* Retz.*, Pogostemon cablin* (Blanco)Benth., GUF	Invigorate resuscitation and resolve phlegm, calm, and soothe the nerves	[2]
Jiannao Capsule	CDM*, Lycium barbarum* L.*, Alpinia oxyphylla* Miq.*, Ziziphus jujuba* Mill. var. *spinosa* (Bunge)*Hu ex* H. F. *Chou,* SCB*, Platycladus orientalis*(L.)*Franco,* SUC, *Dens* Draconis., AES, BTM, *Polygala tenuifolia* Willd., GUF, *Acorus tatarinowii Schott*, GB, CMR, Fe_2_O_3,_ ADS, PGM, *Dioscorea opposita* Thunb., SMB	Nourishes the kidney, nourishes the brain, nourishes the blood, and calms the nerves	[2]
Pediatric Jindan Tablets	PLO*, Arctium lappa* L., *Mentha haplocalyx* Briq., STE, *Tamarix chinensis* Lour., NTC, SDS, *Isatis indigotica* Fort., SNH, RGL, PLP, C_10_H_18_O, CRB, *Fritillaria cirrhosa* D.*Don,* AES, PTT, *Peucedanum praeruptorum Dunn, Platycodon grandiflorum* (Jacq.)A.DC., HgS, URMH, GB, BBL, STL, *Akebia quinata* (hunb.)Decne., CAL, GUF	Dispel wind and phlegm, clear away heat, and detoxify	[2]
Baolong,Pill	*Mentha haplocalyx* Briq.*, ADBH, Perilla frutescens* (L.) Britt.*, Pogostemon cablin* (Blanco)Benth., SDS, APM, STE, *Ligusticum chuanxiong* Hort.*, PCW, AMK, Dioscorea opposita* Thunb., CRB, *Amomum villosum* Lour.*, Piper longum* L.*, Magnolia officinalis* Rehd. *et* Wils., ALD, CRL, *Santalum album* L., GB, BML, BTM, TAG, PTT, [Al_4_(Si_4_O_10_) (OH)_8_•4H_2_O], *Terminalia chebula* Retz., HgS, PLP	Eliminate wind and phlegm, invigorate the spleen and stomach	[2]
Babao Jingfeng granule	BTDG*, Scutellaria baicalensis Georgi, GJE, BTM, Fritillaria cirrhosa* D.*Don, vermiculite Schist seu Hydrobiotite Schist,* AES, GB, URMH, SDS, BMK, *Pteria martensii* (Dunker)*, Dens* Draconis., PCW, ECT, ASG, *Mentha haplocalyx* Briq., MBF	To expel wind and phlegm, reduce fever and suppress convulsions	[2]
Jingtongling Medic,inal Liquor	RGL, PMT, PLP, *Sesamum indicum* L.*, Lycium barbarum* L.*, Drynaria fortunei* (Kunze) J.Sm., CBJS, *Viscum coloratum* (Komar) *Nakai*, AMBVM, PGM, *Dioscorea opposita* Thunb.*, Cervus nippon Temminck,* ADS, SMB, ABB, BCB, CMR, GB, PLO, *Homalomena occulta* (Lour.)*Schott, Elaphe taeniura Cope, Illicium difengpi* K. I. B. et K.I.M., CCO, CCP, CSN, MBF, GUF	Nourish liver and kidney, promote blood circulation, and relieve pain	[2]

**Table 2:** Phenolic compounds containing a benzene ring.

No	Name	Ref
1	Vanillyl alcohol	[34]
2	Vanillin	[34]
3	4-Hydroxybenzyl alcohol	[35]
4	4-hydroxybenzaldehyde	[36]
5	3,4-Dihydroxybenzaldehyde	[24]
6	p-hydroxybenzyl ethyl ether	[37]
7	4-hydroxybenzyl methyl ether	[38]
8	Dimethyl phthalate	[39]
9	Benzyl alcohol	[40]
10	Vanillic acid	[40]
11	1-furan-2-yl-2-(4-hydroxyphenyl) -ethanone	[41]
12	5-(4-hydroxylbenzyloxymethyl)-furan-2-carbaldehyde	[41]
13	gastrodin A	[42]
14	p-methoxybenyl ethyl ether	[33]
15	p-hydroxybenzenemethanol-*β*-D-glucopyranoside	[43]
16	p-methylphenyl-1-O-D-glucopyranoside	[40]
17	3,5-Dimethoxy benzoic acid-4-O-*β*-D-glucopyranoside	[44]
18	4-hydroxybenzyl-*β*-D-glucopyranoside	[39]
19	p-ethoxymethylphenyl-1-O-*β*-D-glucopyranoside	[39]
20	4-hydroxbenzylmethyl ether	[37]
21	Gastrodin	[38]

**Table 3:** Phenolic compounds containing two or more benzene rings.

No	Name	Ref
22	4,4′-Dihydroxydiphenyl methane	[39]
23	4-Hydroxybenzyl ether	[38]
24	4-(4′-hydroxybenzyl-oxy) benzyl methyl ether	[37]
25	2,2′methylene-bis(6-tert-butyl-4-methylphenl)	[44]
26	Gastrol A	[24]
27	p-Hydroxybenyloxy benzlalcohol	[44]
28	4, 4′–Dihydroxybenzyl sulfoxide	[44]
29	4-[4′-(4″-hydroxybenzyloxy)benzyloxy]benzyl methyl ether	[38]
30	Gastrodamine	[44]
31	2, 4-bis(4-hydroxybenzyl) phenol	[38]
32	4-hydroxy-3-(4′-hydroxybenzyl) benzyl alcohol	[45]
33	bis-(4-hydroxybenzyl)sulfide	[46]
34	4, 4′-dihydroxybenzyl sulfone	[47]
35	4-hydroxybenzyl vanillyl ether	[48]
36	4-{{4-[4-(methoxymethyl)phenoxy]benzyl} oxy} benzylmethyl ether	[48]
37	(4-hydroxy-3-(4-hydroxybenzyl) benzylmethyl ether	[48]
38	2-(4-hydroxy-3-(4-hydroxybenzyl)benzyl)-(methoxymethyl)phenol	[49]
39	2-(4-hydroxy-3-(4-hydroxy-3-(4-hydroxybenzyl)benzyl)benzyl)-4-(methoxymethyl)phenol	[49]
40	2-(4-hydroxy-3-(4-hydroxybenzyl)benzyl)-4-(4-hydroxybenzyl)phenol	[49]

**Table 4:** Organic acids and lipids.

No	Name	Ref
41	4,4′-Methylenebis(2-(4-hydroxybenzyl)phenol)	[49]
42	(+)-L-[S-(4-Hydroxybenzyl) cysteinylglycine	[18]
43	(−)-(SS)-*γ*-L-Glutamyl-L-[S-(4-hydroxybenzyl)]cysteinylglycine sulfoxide	[17]
44	Ethyl(−)-(SS)-*γ*-L-glutamyl-L-[S-(4-hydroxybenzyl)]cysteinylglycinatesulfoxide	[18]
45	(−)-(RS)-*γ*-L-Glutamyl-L-[S-(4-hydroxybenzyl)]cysteinylglycine sulfoxide	[17]
46	Ethyl(−)-(RS)-g-L-glutamyl-L-[S-(4-hydroxybenzyl)] cysteinylglycinate sulfoxide	[18]
47	(−)-*γ*-L-[N-(4-Hydroxybenzyl)]glutamyl-L-[S-(4-hydroxybenzyl)]cysteinylglycine	[17]
48	Gastronucleoside	[18]
49	Methyl(−)-*γ*-L-glutamyl-L-S-(4-hydroxybenzyl) cysteinylglycinate	[42]
50	4-(methoxymethyl) phenyl-1-O-*β*-D-glucopyranoside	[33]
51	1-furan- 2-yl-2-(4-hydroxy-phenyl)-ethane–1,2-dione	[50]

**Table 5:** Steroids and their glycosides.

No	Name	Ref
52	Palmitic acid	[40]
53	Citric acid	[44]
54	Gastrol	[37]
55	Parishin	[35]
56	Amber acid	[44]
57	trans-3-Phenylacrylic acid	[44]
58	6-Methyl citrate	[44]
59	Citric acid monomdtyl ester	[44]
60	tri-[4-(*β*-D-glucopy-ranosyloxy) benzyl]citrate	[36]
61	1,2-bis[4-(*β*-D-glucopyranosyloxy) benzyl]citrate	[51]
62	1, 3-bis[4-(*β*-D-glucopyranosyloxy) benzyl]citrate	[52]
63	Parishin D	[53]
64	Parishin E	[3]
65	3-Hydroxybenzoic acid	[40]
66	Syringate	[24]
67	Protocatechuic acid	[3]

**Table 6:** Other categories.

No	Name	Ref
68	*β*-daucosterol	[44]
69	Stigmasterol	[40]
70	Stigmastane-3*β*, 5*α*, 6*β*-triol	[44]
71	*β*-sitosterol	[40]
72	Sucrose	[43]
73	*β*-sitosterol glucoside	[40]
74	cirsiumaldehyde	[44]
75	5-hydroxymethyl furfural	[40]
76	adenosine	[38]
77	N6-(4-hydroxyzenzyl)adenosine	[37]
78	Adenosine glucoside	[3]
79	p-hydroxybenzyl guanosine	[24]
80	7, 8-dimethyl benzo[g]pteridine-2, 4-(1H, 3H)-dione	[3]
81	1-furan-2-yl-2-(4-hydroxy-phenyl)-ethane-1, 2-dione	[46]
82	s-(4-hydroxybenzyl)-giutathione	[38]
83	L-pyroglutamicacid	[54]
84	3, 5-dihydroxy-1, 4-phenanthraquinone	[3]
85	2-(2-(((5-methyl-1,3,4-thiadiazol-2-yl)methyl)amino)ethyl)isoquinoline-1,3(2H, 4H)-dione	[3]
86	(3R,4S,5R)-2-(6-((4-hydroxybenzyl)amino)-9H-purin-9-yl)-5-(hydroxymethyl)tetrahydrofuran-3,4-diol	[3]
87	Benzoic acid	[55]
88	Stearic acid	[55]
89	Hentriacontanoic acid	[55]
90	Dotriacontanoic acid	[55]
91	oxiran-2-ylmethyl docosanoate	[55]
92	Styrene	[56]
93	Benzaldehyde	[56]
94	2-pentylfuran	[56]
95	1-methyl-4-(prop-1-en-2-yl)cyclohex-1-ene	[56]
96	2-phenylacetaldehyde	[56]
97	2-phenylacetaldehyde	[56]
98	(10E, 13E)-2-ethoxynonadeca-1,10,13-triene	[56]

**Table 7:** Pharmacological effects.

Pharmacological effects	Detail	Extracts/Compounds	Minimal active concentration/Dose	In Vitro/In Vivo	Ref
Central nervous system	Hypnosis and sedation	Fresh gastrodia	3 g/kg	In Vivo	[57]
		Gastrodin	50 mg/kg	In Vivo	[58]
		Gastrodia	1.2 g/kg	In Vivo	[59]
		Gastrodia	1.5 g/kg	In Vivo	[60]
	Anti-Parkinson's disease	Gastrodia	5 g/kg	In Vivo	[61]
		Gastrodia	5 g/kg	In Vivo	[62]
		Gastrodia	0.4 g/kg	In Vivo	[63]
		Gastrodia	7.2 g/kg	In Vivo	[64]
		Gastrodin	50 mg/kg	In Vivo	[65]
		Gastrodin	100 mg/kg	In Vivo	[66]
		Saponins	1.5 g/kg	In Vivo	[67]
		Gastrodia Gastrodin	2 g/kg 100 mg/kg	In Vivo In Vivo	[68] [69]
	Antidepressant	Gastrodin	100 mg/kg	In Vivo	[70]
		Gastrodin	400 mg/kg	In Vivo	[71]
	Anticonvulsant	Stalks	500 mg/kg	In Vivo	[72]
		Seed	225 mg/kg	In Vivo	[73]
	Antivertigo	Gastrodin	600 mg/d	In Vivo	[74]
		Gastrodin	600 g/d	In Vivo	[75]
		Gastrodin	600 g/d	In Vivo	[76]
	Analgesia	Gastrodin	100 g/kg	In Vivo	[77]
	Antiepileptic	Gastrodin	0.37 ml/kg	In Vivo	[78]
		Gastrodin	50 mg/kg	In Vivo	[79]
		Gastrodin Gastrodin	200 mg/kg 200 mg/kg	In Vivo In Vivo	[80] [81]
		Gastrodin	60 mg/kg	In Vivo	[62]
	Protects nerve cells	Gastrodia	0.5 g/kg	In Vivo	[82]

Cardiovascular system	Protects cardiomyocytes	Gastrodin	10 *μ*mol/L	In Vitro	[83]
		Gastrodin	50 mg/kg	In Vivo	[74]
		Gastrodin	20 mg/L	In Vitro	[21]
		Gastrodin	50 mg/kg	In Vivo	[84]
	Antihypertension	Gastrodin	0.6 g/d	In Vivo	[85]
		Gastrodia	10 mL/kg	In Vivo	[72]
		Extract G2	1.2 mg/ml	In Vivo	[86]
	Antiplatelet aggregation and antithrombosis	Gastrodin	20 mg/kg	In Vivo	[87]
		Gastrodin	7.5 mg/kg	In Vivo	[88]
		Gastropodol	8 mg/kg	In Vivo	[89]
	Promotes angiogenesis	Gastrodin	10 *μ*g/kg	In Vivo	[90]
		Ethanol extract	0.94 g/kg	In Vivo	[35]
		*GB*	200 mg/kg	In Vivo	[91]

Skeletal system		Gastrodin	5 mg/kg	In Vivo	[45]
		Gastrodin	5 g/kg	In Vivo	[92]
		Gastrodin	25 mg	In Vivo	[93]
		Ethanol extract	100 mg/kg	In Vivo	[94]

Digestive system		Aqueous extract	0.5%	In Vitro	[95]
		Gastrodin	0.1 *µ*M	In Vitro	[96]
		Gastrodia	25 g/kg	In Vitro	[97]

Urinary system		Gastrodin	0.30 g/kg	In Vitro	[40]
		Bis-(4-hydroxyphenyl)methane, benzyl alcohol 4-Hydroxybenzaldehyde	12.5 mg/kg	In Vitro	[98]
Respiratory system		Aqueous extract	10 ml/kg	In Vitro	[99]
Strengthens immunity		Gastrodin	400 mg/kg	In Vivo	[100]

Others	Antioxidant	Gastrodin	50 mg/ml	In Vitro	[53]
		Gastrodia	20 mg/kg	In Vivo	[52]
		Gastrodin	2 mL/d	In Vivo	[77]
	Treatment of deafness and tinnitus	Gastrodin	0.6 mL/d	In Vivo	[101]

## Data Availability

The data that support the findings of this study are available from scientific papers, books, and dissertations concerning GB. Some dissertations and scientific databases were used, including Baidu Scholars, Science Net, Weipu, Wanfang, and CNKI.

## Abbreviations

ACL: *Areca catechu* L
AD: Alzheimer's disease
ADP: Adenosine diphosphate
AGS: *Acanthopanax gracilistylus* W.W.Smith
AHFSVM: *Asarum heterotropoides* Fr. Schmidt var. *mandshuricum* (Maxim.)Kitag
AKP: *Amomum kravanh Pierre* ex Gagnep
AKR: *Aconitum kusnezoffii* Reichb
ALD: *Aucklandia lappa* Decne
ALL: *Arctium lappa* L
AMBVM: *Astragalus membranaceus* (Fisch.) Bge. var. *mongholicus (*Bge.*)Hsiao*
AMK: *Atractylodes macrocephala* Koidz
APM: *Angelica pubescens* Maxim.f. *biserrata Shan et Yuan*
ASD: *Angelica sinensis* (Oliv.)*Diels*
ASG: *Aquilaria sinensis* (Lour.)*Gilg*
AV: *Artemisia argyi* Lévl. et Van
A*β*: Beta-amyloid
BBL: *Bubalus bubalis Linnaeus*
BCB: *Boswellia carterii* Birdw
BCD: *Bupleurum chinense* DC
BMK: *Buthus martensii Karsch*
BML: *Bombyx mori Linnaeus*
BOV: *Bovidae*
BSF: *Beckmannia syzigachne* (Steud.) Fern
BTDG: *Bos taurus domesticus Gmelin*
BTM: *Bambusa textilis McClure*
CAB: *Cuscuta australis* R. Br
CAL: *Citrus aurantium* L
CBJS: *Cibotium barometz*(L.)J.Sm
CCLP: *Cinnamomum camphora*(L.)*Presl*
CCO: *Clematis chinensis Osbeck*
CCP: *Cinnamomum cassia Presl*
CDM: *Cistanche deserticola* Y. C. Ma
CGT: *Citrus grandis* “Tomentosa”
CME: *Commiphora myrrha* Engl
CMR: *Chrysanthemum morifolium* Ramat
COP: *Coptis chinensis* Franch
CRB: *Citrus reticulata Blanco*
CRL: *Cyperus rotundus* L
CSN: *Chaenomeles speciosa* (Sweet)*Nakai*
CUMS: Chronic unpredictable stress model
D2: Dopamine receptor 2
DA: Dopamine
DDB: *Daemonorops draco* Bl
ECT: *Eugenia caryophyllata* Thunb
EPO: Eosinophil peroxidase
ESS: *Ephedra sinica Stapf*
ETC: *Elaphe taeniura Cope*
EUO: *Eucommia ulmoides* Oliv
GABA-T: 7-Aminobutyrate transaminase
GB: *Gastrodia elata* BI
GJE: *Gardenia jasminoides Ellis*
GTCS: Generalized tonic-clonic seizure
GUF: *Glycyrrhiza uralensis* Fisch
HMGB1: High mobility group box-1
HO-1: Heme oxygenase-1
Kepa1: Kelch-like epoxylopropylamine-related protein 1
LCHSMB: *Ligusticum chuanxiong* Hort. *Salvia miltiorrhiza* Bge
LSO: Ligusticum sinense Oliv
MBF: *Moschus berezovskii Flerov*
MCAO/R: Middle cerebral artery occlusion/reper-fusion
MCS: Minimal clonic seizure
MPTP: Mitochondrial permeability transition pore
NTC: *Notopterygium incisum* Ting ex H.T.Chang
PAE: *Pheretima aspergillum* (E.Perrier)
PAF: Platelet activating factor
p-AKT: Serine-threonine protein kinase
PCP: *Picrorhiza scrophulariiflora Pennell*
PCW: *Poria cocos*(Schw.) Wolf
PD: Parkinson's disease
PGM: *Panax ginseng* C. A. Mey
PKCH: *Polygonatum kingianum* Coll.et Hemsl
PLO: *Pueraria lobata*(Willd)Ohwi
PLP: *Paeonia lactiflora* Pall
PMT: *Polygonum multiflorum* Thunb
PNC: *Panax notoginseng* (Burk.)F.H.Chen
PTT: *Pinellia ternata* (Thunb.)
QIL: *Quisqualis indica* L
RGL: *Rehmannia glutinosa* Libosch
RPL: *Rheum palmatum* L
SCB: *Schisandra chinensis*(Turcz.)Baill
SDS: *Saposhnikovia divaricata* (Turcz.) Schischk
SIRT1: Silent Information Regulator 1
SMB: *Salvia miltiorrhiza* Bge
SNH: *Scrophularia ningpoensis* Hemsl
SOD: Superoxide dismutase
SSMK: *Scolopendra subspinipes mutilans* L. Koch
STE: *Schizonepeta tenuifolia* Eriq
STL: *Saiga tatarica Linnaeus*
SUC: *Succinum*
TGE: *Typhonium giganteum* Engl
TGF-*β*: transforming growth factor-*β*
Th17: Thelper cell 17
URMH: *Uncaria rhynchophylla* (Miq.)Miq. ex Havil
VEGF-A: Vascular endothelial growth factor-A
VEGFR-2: Vascular Endothelial Growth Factor Receptor.
